# Durable superoleophobic–superhydrophilic fabrics with high anti-oil-fouling property†

**DOI:** 10.1039/c8ra04645j

**Published:** 2018-07-30

**Authors:** Hua Zhou, Hongxia Wang, Weidong Yang, Haitao Niu, Xin Wei, Sida Fu, Shuai Liu, Hao Shao, Tong Lin

**Affiliations:** Institute for Frontier Materials, Deakin University Geelong VIC 3216 Australia hong.wang@deakin.edu.au tong.lin@deakin.edu.au; Future Manufacturing Flagship, CSIRO Clayton South VIC 3169 Australia; School of Mechanical and Electric Engineering, Soochow University 215000 China

## Abstract

Although a number of methods have been reported for the preparation of superoleophobic–superhydrophobic surfaces, a challenge still remains in preparing a surface showing simultaneous superoleophobicity and superhydrophilicity. Herein, we demonstrate a novel strategy for preparing a simultaneously superhydrophilic–superoleophobic surface on cotton fabrics. A wet chemical coating method was employed to apply an oligomer which consists of a fluorinated alkyl and a PEG-phosphate hydrophilic moiety, silica nanoparticles and fluoroalkyl silane, onto fabric substrates. The treated fabrics exhibited both superoleophobicity and superhydrophilicity with a contact angle over 150° for oil fluids (surface tension > 27 mN m^−1^) but 0° for water. Water can spread into the fabric matrix within 2 seconds. The superhydrophilic–superoleophobic fabric had excellent superoleophobicity no matter whether it was at the dry state in air, pre-wetted with water, or in underwater conditions. When being contaminated forcedly with oil or oil-free dirt, the fabric can be easily cleaned up with water without using any detergent and chemical agent. Superoleophobic–superhydrophilic surfaces may provide an alternative and feasible way for anti-fouling applications.

## Abstract 2

Although a number of methods have been reported for the preparation of superoleophobic–superhydrophobic surfaces, a challenge still remains in preparing a surface showing simultaneous superoleophobicity and superhydrophilicity.

## Graphical abstract

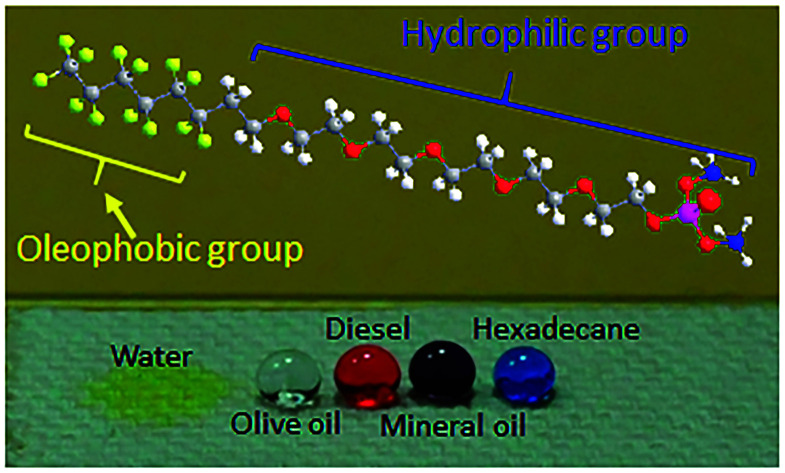

## Introduction

Superoleophobic surfaces conventionally show a superhydrophobic feature because oil fluids often have lower surface tension than water. According to the Young's equation *γ* cos *θ* + *γ*_sl_ = *γ*_s_, when a liquid drop is placed on a solid surface, the equilibrium contact angle (*θ*) is determined by the liquid surface tension (*γ*), solid–gas interface tension (*γ*_sg_) and solid–liquid interface tension (*γ*_sl_). Lowering the *γ* and *γ*_sl_ would lead to a decrease of *θ*. A surface that is repellent to oil fluid usually has stronger repellency to water, and as a result the static contact angle for water (*θ*_water_) is generally larger than that for oil fluid (*θ*_oil_).^1–3^

Several papers have reported a special wettability phenomenon where the surface shows a larger contact angle for oil fluid than water, *i.e. θ*_oil_ > *θ*_water_.^4–16^ In the early papers, this unusual property was found in the oleophilic region where *θ*_oil_ < 90°,^4–6,8,12^ and it was attributed to intercalation between hydrophilic and oleophobic constituents which allows water molecules to penetrate the oleophobic zone and spread in hydrophilic domains. A recent mini-review summarized the research progress in this area.^15^

Recently, superoleophobic–superhydrophilic surfaces have been reported,^16–20^ though most of the surfaces reported have either unstable hydrophilicity or an ageing issue. Some of the surfaces were initially superamphiphobic and had to be activated by water, a special chemical, or UV radiation before they showed superhydrophilicity.^19–21^ Xu *et al.*^19^ from our group reported a superhydrophilic–superoleophobic surface that was prepared by treatment of a superamphiphobic surface in ammonia vapor. Tang *et al.*^20^ prepared a superhydrophilic–superoleophobic surface by UV light treatment of a superamphiphobic surface. Li *et al.*^16^ reported a superoleophobic–superhydrophilic surface prepared from a solution containing a commercial fluorine-surfactant (capstone FS-50) and TiO_2_ nanoparticles. In those studies, novel applications of the superhydrophilic–superoleophobic surfaces for water/oil separation were also revealed. Nevertheless, it remains a challenge to prepare stable superoleophobic–superhydrophilic surfaces.

In contrast to the above-mentioned simultaneously superhydrophilic–superoleophobic surfaces, surfaces with simultaneous hydrophilicity/underwater superoleophobicity are very common. For a solid surface with superhydrophilicity in air, no matter whether it is oleophobic or oleophilic, it shows superoleophobicity in underwater condition, because once the surface is wetted with water, the actual wettability is governed by the water layer on surface. Underwater superoleophobic surfaces are widely found in aquarium creatures. A famous example is fish's scales which always show a large underwater contact angle and strong fouling resistance to oil fluids.^22–25^ Such bio-mimicking surfaces show potential applications for self-cleaning,^26,27^ underwater antifouling,^28^ oil/water separation,^29,30^ and pollution resistance.^31^ The fish-scale-inspired underwater superoleophobic surfaces are prepared mainly from hydrophilic–oleophilic surfaces.^25,32,33^ Hydrophilic–superoleophobic surfaces for fouling resistant applications have been less reported.

In this study, we demonstrate a novel strategy for making a stable, simultaneously superhydrophilic–superoleophobic (SHI–SOP) surface on cotton fabrics. A two-step wet-chemical coating method was employed to apply an oligomer which consists of a low surface energy fluorinated alkyl and a PEG-phosphate hydrophilic moiety, silica nanoparticles and fluoroalkyl silane, onto fabric substrate. After coating treatment, the fabric showed stable superhydrophilicity with water contact angle of 0°. Water droplet can spread into the fabric matrix within 2 seconds. The fabric also showed superoleophobicity with a stable contact angle over 150° to oils (surface tension > 27 mN m^−1^) no matter whether it was at dry state in air, pre-wetted with water, or in underwater environment. The coating was durable and can withstand strong acid/base solutions, repetitive abrasion, and longtime immersion in water. We further showed that the coated fabric had excellent fouling resistance. When being contaminated forcedly with oils or non-oil dirt, the fabric can be cleaned easily with water without using any detergent and chemical.

## Results and discussion

**Fig. 1:**
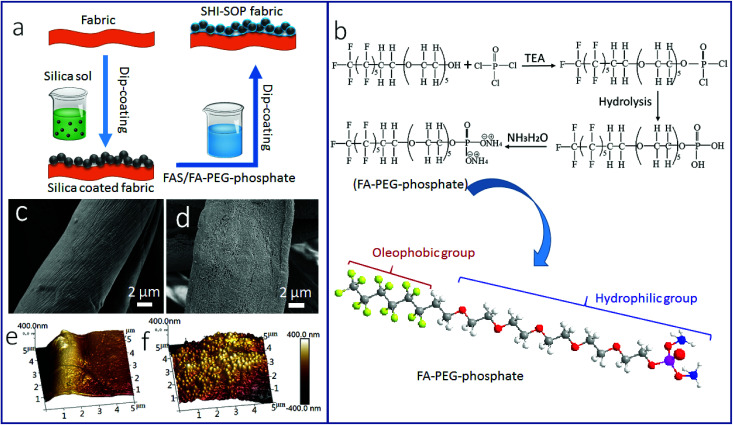
(a) Schematic illustration of the procedure for coating treatment, (b) chemical reactions to synthesize FA-PEG-phosphate and the chemical structure of the synthesised FA-PEG-phosphate. (c and d) SEM images of: (c) uncoated cotton, (d) silica NP/FAS/FA-PEG-phosphate coated cotton. (e and f) AFM images of: (e) uncoated cotton, and (f) silica NP/FAS/FA-PEG-phosphate coated cotton.

Fig. 1a illustrates the procedure for SHI–SOP coating treatment of cotton fabrics. Two coating solutions, one containing silica nanoparticles (average size around 200 nm, see the AFM image and size distribution in Fig. S1, ESI†) and another containing a small molecular oligomer (FA-PEG-phosphate) and fluorinated alkyl silane (FAS), were applied in sequence onto the fabric using a dip-coating method. Fig. 1b shows the synthesis route of FA-PEG-phosphate. The chemical structure of FA-PEG-phosphate was verified by nuclear magnetic resonance (NMR, see the spectrum in Fig. S2, ESI†).

Fig. 1(c and d) shows the SEM images of the fabric before and after coating treatment. A particulate morphology can be clearly seen on fiber surface after coating treatment (Fig. 1d). Atomic force microscope (AFM) imaging was used to measure the surface roughness of the cotton fibers (Fig. 1e and f). The uncoated cotton fiber had a root mean squared (RMS) roughness of 302 nm (Fig. 1e). After coating treatment, the RMS roughness increased to 676 nm (Fig. 1f). The TEM imaging indicated that the coating was around 500–600 nm in thickness (see in Fig. S3, ESI†), suggesting a multilayer molecular structure.

**Fig. 2:**
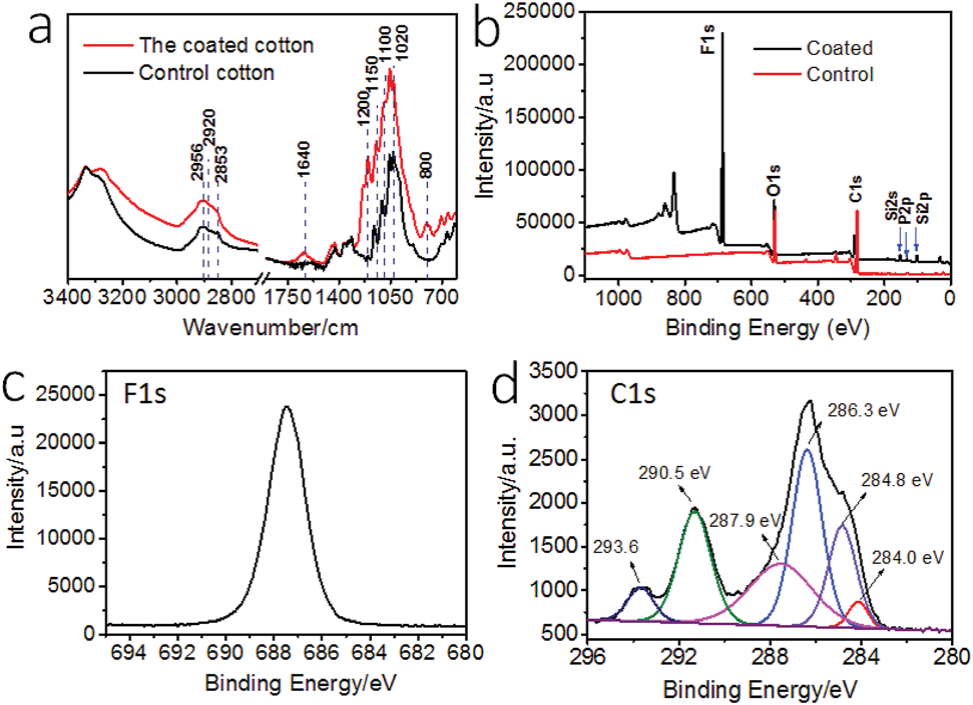
(a) FTIR spectra of the uncoated and coated cotton, (b) XPS survey of the uncoated and the coated fabrics, (c) high resolution F 1s spectrum of coated cotton, (d) high resolution C 1s spectrum and curved-fitted results of coated cotton.

The surface chemistry of the cotton fabric before and after coating treatment was examined by Fourier transform infrared (FTIR) and X-ray photoelectron spectroscopy (XPS). Fig. 2a show the FTIR spectra. After coating treatment, new peaks occurred at 1200 cm^−1^ and 1150 cm^−1^, which can be assigned to P=O and C–F stretching vibrations. The peaks around 700 cm^−1^ were assigned to C–F stretching. The increased intensity at ∼1100 cm^−1^ corresponded to C–F and Si–O stretching vibrations. The peak at 1020 cm^−1^ was assigned to P–O stretching. The new vibration band at 800 cm^−1^ was assigned to Si–O–Si stretching. The band at 1640 cm^−1^ was assigned to the O=P–OH and Si–OH stretching. The increased peaks at 2956 cm^−1^, 2920 cm^−1^, and 2853 cm^−1^ were assigned to P–OH stretching vibrations.

Fig. 2b shows the XPS survey spectra of the fabrics. The coated fabric showed elements F, Si, and P on surface. The binding energy at 830 eV is F KLL Auger peak. The high resolution XPS F 1s, and C 1s and curved-fitted C 1s result are shown in Fig. 2c and d. The peaks at binding energy 687.5 eV was attributed to the F 1s. The peaks at 284.0 eV and 286.3 eV were assigned to C–Si and C–O groups, and the peak at 284.8 eV was assigned to C–C and C–H groups. The peaks at 287.9 eV corresponded to carbonyl C=O and O–C–O. The peaks at 293.6 eV and 290.5 eV corresponded to CF_3_ and CF_2_. In addition, Si 2p and P 2p also showed peaks at 102.0 eV and 133.0 eV, assigned to Si–O group and –PO_4_ in phosphate, respectively (Fig. S4†).

**Fig. 3:**
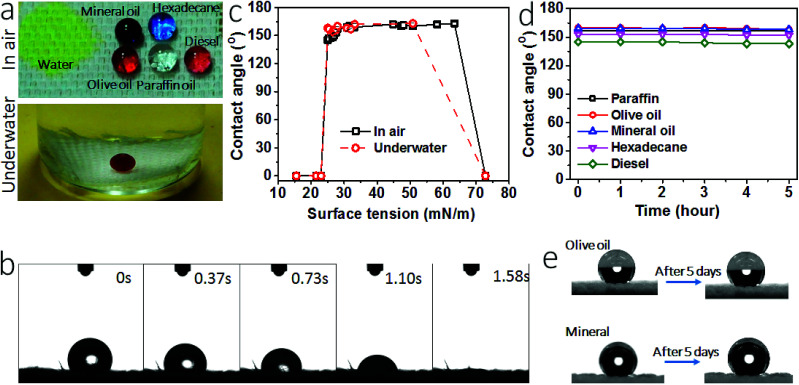
(a) Photos of liquid droplets on the coated fabric in air (10 μL each drop, yellow water, clear paraffin oil, purple mineral oil, red olive oil (left), red diesel (right), blue hexadecane, a small amount of dyes were used, which had no influence on the wettability) and underwater (red liquid drop is DCE, 15 μL), (b) variation of water droplet on the coated cotton fabric with time, (c) dependency of CA on the surface tension of liquids, (d) water and oil CA changes with time for the coated fabric, (e) photos to show oil droplets before and after 5 days of leaving on the coated fabric.

Fig. 3a shows the wetting properties of the coated fabric. When clear paraffin oil, red-colored olive oil, purple-colored mineral oil, light red-colored diesel and blue-colored hexadecane were dropped onto the fabric, they all formed sphere-like droplets. The contact angles (CAs) of the fabric for these oil fluids were 157°, 160°, 159°, 145° and 153°, respectively, suggesting that the coated fabric has a superoleophobic surface. The oil sliding angles of the coated fabric were measured, being 16° and 19° for olive oil and mineral oil. For the oils with surface tension below 27.5 mN m^−1^, they sick on the surface without rolling off, suggesting a much larger sliding angle. In contrast, the uncoated cotton fabric was oleophilic and had a CA of 0° for all these oil fluids.

Water contact angle and the time for water drop to spread completely into fabric matrix were used to characterize hydrophilicity. When yellow-colored water was dropped onto the coated fabric, it spread completely into the fabric matrix in 1.58 seconds (Fig. 3b, also see the spreading process in Video S1, ESI†), indicating strong affinity of the coated fabric to water. The fabric had a water contact angle of 0°. For comparison, we summarized water contract angle and spreading time data of the superhydrophilic surfaces reported in literature (Table S1, ESI†). Indeed, our coated fabric showed a similar superhydrophilicity to these surfaces.

Apart from the wetting properties in dry state, the wettability in underwater condition was also examined. Fig. 3a also shows 1,2-dichloroethane (DCE) droplet on the coated fabric in water. In such a fully wetted state, DCE stayed stably in sphere-like droplet on the fabric surface, with an underwater CA (CA_uw_) of 170°. To further prove the excellent underwater oil repellency, we immersed the coated fabric in oil fluid. A plastron layer can be clearly seen between the fabric and the oil interface (Fig. S5†). After 5 hours, this plastron layer was still there and the fabric was non-wetted as well, suggesting the excellent underwater superoleophobicity. When water was dropped on the fabric in oil, however, it spread into the fabric matrix rapidly (Fig. S6, Video S2, ESI†).

A series of liquids with different surface tension (*γ*) values in the range from 15.5 mN m^−1^ to 72.8 mN m^−1^ were used to probe the oil repellency. Fig. 3c shows the relationship between the liquid surface tension and CA (see the data in Table S2, ESI†). At dry state, the coated fabric had a CA above 150° for the liquids with a surface tension in the range between 27–63 mN m^−1^. At underwater state, the oil CA_uw_ was above 150° for the oil fluids with surface tension in the range of 27–50.8 mN m^−1^ (see details in Fig. S7 and Table S3, ESI†). However, at both dry and underwater states the fabric showed a contact angle of 0° for water and the oil liquids of surface tension below 23 mN m^−1^. Such CA–*γ* relationships are unusual and differ to the previously reported superamphiphobic fabrics.^34,35^

Fig. 3d shows the change of CA with time. All oil drops can stay stably on the coated fabric for 5 hours except for water. The oil drops can even stayed on the fabric for 5 days in ambient condition (see the photo in Fig. 3e). These results indicate that the coated fabric has a stable superoleophobicity. We also tested the long time stability of the coating. After being stored in ambient condition for 1 month, the fabric still kept the SHI–SOP feature unchanged (Fig. S8, ESI†). In addition, the coated fabric maintained stable SHI–SOP property in water. After immersing in water for 24 hours, the fabric still kept the original SHI–SOP property (Fig. S9, ESI†). These results suggest that both the superoleophobic and the superhydrophilic properties are very stable without any ageing issue.

**Fig. 4:**
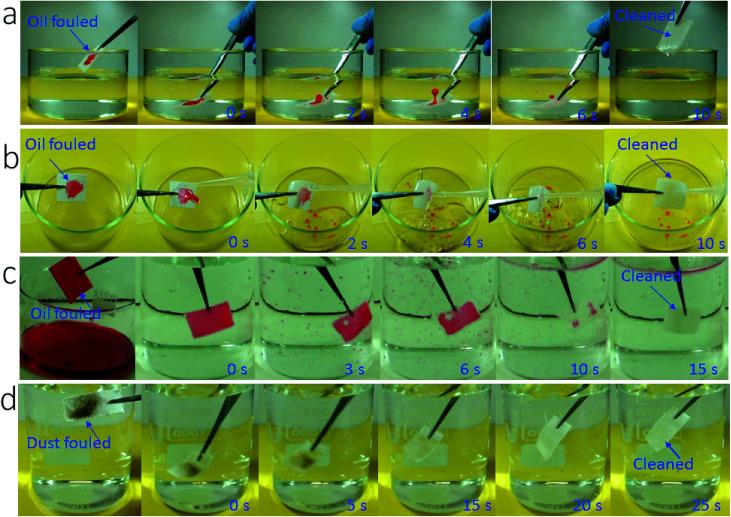
Snapshots taken from videos to show cleaning of oil-contaminated fabrics with tap water, (a) pre-contaminated with a small amount of olive oil (50 μL, the fabric was just immersed in water without shaking), (b) pre-contaminated with a small amount of olive oil (50 μL, the fabric was rinsed with water), (c) olive oil fully fouled fabric after immersing and shaking in water, (d) dust-contaminated cotton fabric in water.

More interestingly, the coated fabric exhibited strong fouling resistance to oil fluids (*γ* = 27–50.8 mN m^−1^). To demonstrate this, we applied oils (*e.g.* olive oil, diesel and mineral oil) on the coated fabric samples and then rubbed the fabrics with hand to ensure that the oils spread within the fabric matrix to fill all the porous spaces and contact closely with the fibers. Then we used two separate methods to test the fouling resistance: immersing in water and rinsing with tap water. When the oil-fouled fabrics were immersed in water, the oil layer started shrinking into a droplet and in 10 seconds it detached completely from the fabric surface (Fig. 4a, also see Video S3 and Fig. S10, ESI†). The oil fouled fabric can also be cleaned easily by rinsing with tap water (see Fig. 4b and Fig. S11 and Video S4, ESI†).

To further test the fouling resistance, we immersed the coated fabric completely in olive oil. By vacuuming the sample-containing oil fluid to remove the plastron layer, oil filled into the porous space of the coated fabric and spread on each individual fibers. Then the contaminated fabric was immersed in water. When the fabric was shaken gently with hand, the oil floated off the fabric leaving a completely clean fabric (Fig. 4c and Video S5, ESI†).

In addition, the SHI–SOP fabric showed a strong fouling resistance to oil-free dirt. Soil powder was put onto the SHI–SOP fabric and the fabric was then rubbed to ensure the soil spreading into fabric matrix. The dirt contaminated fabric was then immersed in water with gentle shaking. As seen in Fig. 4d, the dirt detaches from the fabric in 25 seconds, restoring a completely clean surface. This anti-dust-contamination property was attributed to the excellent superhydrophilicity of the fabric, which offers stronger affinity to water than dust powders. As a result, the dust was replaced with water when the contaminated fabric contacted with water, no matter whether the dust was hydrophilic or oily. This is different to superhydrophobic surface, where dust powder has much stronger affinity to water than the superhydrophobic surface.

Here it should be pointed out that conventional superoleophobic and superamphiphobic (*i.e.* being both superoleophobic and superhydrophobic) surfaces, which show superhydrophobicity, typically have anti-oil-fouling features.^35–41^ To find out the difference between the conventional anti-oil-fouling surfaces and our SHI–SOP fabric, we tested a superamphiphobic fabric (see the preparation and test result in Fig. S12, ESI†). The superamphiphobic fabric after being contaminated forcedly with oil cannot be cleaned easily with water due to the strong water repellency.

For comparison, we also tested the fouling resistance of uncoated cotton fabric. It is known that cotton fabric is hydrophilic and oleophilic. Without pre-wetting, cotton is easy to be contaminated with oil due to the oleophilic feature. Once contaminated the oil cannot be removed from the fabric with water alone (Fig. S13 and Video S6, ESI†).

Oil contamination is a widely spreading issue in daily life and industry. Conventional ways to clean up oil contamination from solid surfaces often consume a large volume of water with the aid of detergent, chemical or solvent, usually under the action of mechanical force or heat. Our SHI–SOP coating could offer a solution to clean up fabrics only with water. Not using detergent and chemical will reduce the cost and pollution to the environment. It should be very useful for the areas where the fabrics are often oil-contaminated and required frequent cleaning on a large scale (workwear for instance).

**Fig. 5:**
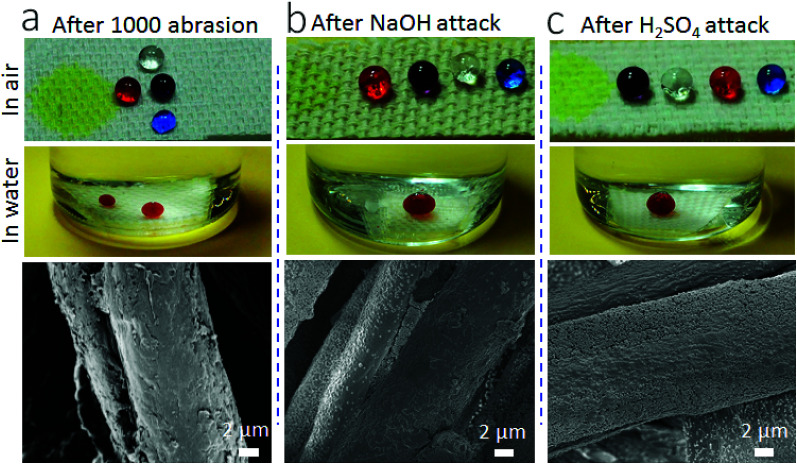
Photos and SEM images of the coated fabric after, (a) 1000 cycles of abrasion, (b) immersing in NaOH solution (pH = 14) for 5 hours followed by rinsing with water and drying at 130 °C for 30 minutes, and (c) immersing in H_2_SO_4_ solution (pH = 1) for 5 hours followed by rinsing with water and drying at 130 °C for 30 minutes (liquid droplets: yellow water, clear paraffin oil, red olive oil, purple mineral oil, and blue hexadecane).

In addition, the coating is durable against repeated abrasion (Fig. 5). After 1000 cycles of Martindale abrasion, the fabric still showed SHI–SOP property (Fig. 5a). The SEM imaging confirmed that some nanoparticles were removed from the surface after abrasion. Even though, the voids left still contribute to large roughness on the surface. The coated fabric was also durable against strong acid/base attacks. After immersing in aqueous NaOH (pH = 14) or H_2_SO_4_ solution (pH = 1) for 5 hours, followed by rinsing with water and drying at 130 °C, the coated fabric still maintained its SHI–SOP property (Fig. 5b and c). There was no morphological change on the coated fiber surface after acid or base treatment.

To probe the role of each coating component in the SHI–SOP function, we conducted a series of experiments, including applying FA-PEG-phosphate only onto fabric (FA-PEG-phosphate concentrations 1–5%), adjusting the weight ratio of FAS/FA-PEG-phosphate in the coating solution, and using surface-hydrophobized silica NPs and surface-hydrophobized cotton fabric (see experimental details in Fig. S14 and S15, ESI†). Our studies have indicated that all the three components are essential for the formation of simultaneously superoleophobic–superhydrophilic surface, and 3.0% FA-PEG-phosphate with a FAS/FA-PEG-phosphate ratio of 1 : 6 (wt/wt) leads to the highest superoleophobicity–superhydrophilicity.

**Fig. 6:**
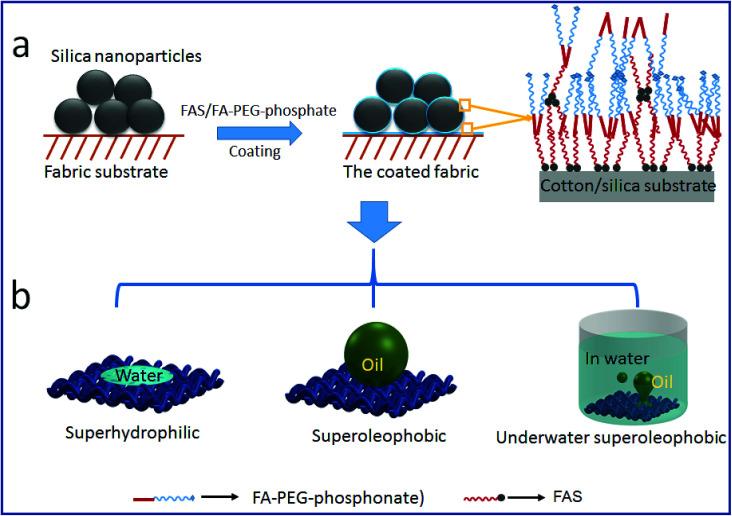
The coating mechanism: (a) the coating procedure and the possible interaction of FAS and FA-PEG-phosphonate on silica and fabric substrate, (b) the wetting properties of the coated fabric.

Fig. 6 schematically illustrates the possible mechanisms for the formation of stable SHI–SOP coating and its oil fouling resistance. FAS comprises a silane head group and a fluoroalkyl chain (length approximately 1.1 nm). FA-PEG-phosphate consists of a fluoroalkyl chain similar to FAS and a hydrophilic PEG-phosphate moiety. The total length of FA-PEG-phosphate molecule is around 3.2 nm with the hydrophilic moiety taking about 2/3 (Fig. S16, ESI†). FA-PEG-phosphate has a much smaller size than silica NP (size around 200 nm), but larger than FAS molecule.

During coating, the silica particles were firstly applied and immobilized onto cotton fibers, and then FAS and FA-PEG-phosphate molecules were applied to the silica NP coated cotton fabric. The FAS molecules can form chemical bonds with silica NPs through silanol groups of the silica NPs and with cotton through the hydroxyl groups of cotton.^42,43^ FAS also has a strong affinity to FA-PEG-PA because they both have a fluorinated alkyl. Therefore, FA-PEG-PA can be immobilized into the coating layer through strong interaction with the FAS attached to the silica NPs and cotton. In this way, the three components aggregated firmly on cotton fibers forming a stably coating layer. Because of the multilayer molecular structure of the coating, FAS molecules could be also embedded in the coating layer through self-bonding between silane heads and interactions between the fluorinated alkyl chains. Fig. 6a illustrates the possible structure of the coating on cotton.

In the coating layer, PEG-phosphate groups aggregated partially and expose to coating surface making the areas hydrophilic. Fluorinated alkyl groups could also expose to the coating surface due to the FAS embedded. In dry state, the scattering low surface energy aggregates to show oleophobicity^44^ and the rough surface formed by silica NPs and the fabric texture enhanced the oil repellency. The co-existence of hydrophilic and oleophobic groups, together with the surface roughness (from coating and fabric texture), results in superhydrophilicity and superoleophobicity (see the illustration in Fig. 6b).

In water, the superhydrophilicity is enhanced because water molecules in the hydrophilic layer increase hydrophilicity. However, the top low surface tension domains are not affected by water. They still have high repellency to oil fluids.

When the coated fabric is forcedly contaminated with oil, the oil molecules have a weak interaction with the coating surface through van der Waals forces.^45^ Water has stronger interaction with the hydrophilic layer, through ion-dipole or hydrogen-bonding interactions for instance.^45,46^ When water molecules approach an oil-contaminated coating surface, they induce the drainage, displacement, and dewetting of oil from the coating surface.

The excellent abrasion durability of the coating on cotton fibers is attributable to the addition of FAS, which functions like a binding agent. The improvement of superhydrophobic coating adhesion by the addition of FAS has been reported by other papers.^43^ FAS can bond to the OH groups on cotton, and the fluorinated alky, and has strong affinity to the FA-PEG-PA fluorinated alkyl chains in the coating layer. Such a coupling effect allows the coating have a reasonable adhesion to the fiber substrate.

## Conclusions

We have demonstrated the preparation of a stable superhydrophilic–superoleophobic surface on cotton fabrics using a wet-chemical coating method. The simultaneously superoleophobic–superhydrophilic fabrics show an excellent anti-oil-fouling ability. When being forcedly contaminated, they can be easily cleaned up with water without using any detergent and chemical agent. Such a stable superoleophobic–superhydrophilic surface property was attributed to the co-existence of hydrophilic and oleophobic functional groups within the coating layer, and strong affinity among the coating materials and with the fiber substrate. Superhydrophilicity–superoleophobicity may provide an alternative and feasible way for anti-fouling applications.

## Methods

### Materials

1*H*,1*H*,2*H*,2*H*-perfluorodecyltriethoxysilane (FAS, C_16_H_19_F_17_O_3_Si), tetraethyl orthosilicate (TEOS), anhydrous diethyl ether, phosphorous oxychloride, fluorinated alkyl-polyethylene glycol (FA-PEG, *M*_w_ ≈ 600), trimethylamine, oil red, oil blue, and oil green were purchased from Sigma-Aldrich and used as received. Ammonium hydroxide (28%) and ethanol were purchased from Chem-Supply Pty Ltd. Commercial cotton fabric (plain weave, 160 g m^−2^, thickness ≈ 510 μm) was obtained from local store.

### Synthesis of FA-PEG-phosphate

Phosphorous oxychloride (POCl_3_, 0.76 g, 5 mol) was added to 20 mL anhydrous diethyl ether under nitrogen protection. A solution of FA-PEG (0.5 g) and triethylamine (1.5 g, 15 mmol) in 25 mL diethyl ether was slowly added, leading to the formation of white precipitate. The solution was kept at room temperature and was allowed to stir under nitrogen for further 2 hours. The resulting white solid (triethylamine hydrochloride salt) was filtered and the cake was washed with 20 mL diethyl ether 3 times. The combined ether solution was dried at ambient for 12 hours to allow the solvent evaporated completed. 20 mL water was added to the obtained paste-like FA-PEG-phosphorous oxychloride product and stirred for 1 h to hydrolyze phosphorous chloride. The cloudy solution was extracted with 3 × 20 mL diethyl ether. After drying with anhydrous magnesium sulfate, the ether was removed by rotary evaporation to give FA-PEG-PA as a very viscous liquid. ^1^H NMR (400 MHz, CDCl_3_): *δ* (ppm) = 2.2–2.5 (m, –CF_2_CH_2_–), 3.5–3.8 (m, –OCH_2_CH_2_O–), 4.1 (m, –CH_2_–O–P–), 7.8 (s, O=P(OH)_2_). ^19^F NMR (376.5 MHz, CDCl_3_): *δ* (ppm) = −81.8 (m, CF_3_), −114.4 (m, –CF_2_CH_2_–), −123–125 (m, –CF_2_–), −127.7 (m, CF_3_CF_2_–). ^31^P NMR (162 MHz, CDC_l3_): *δ* (ppm) = 1 (–O=P(OH)_2_).

### Preparation of FA-PEG-phosphate coating solution

The resulted product was neutralized with diluted ammonia (2 : 1 wt/wt) solution. 1.5 g neutralized FA-PEG-phosphate was then dissolved in 48.5 mL water to make a 3% FA-PEG-phosphate coating solution.

### Synthesis of silica nanoparticle sol

Silica nanoparticles was synthesized according to the literature reported Stober method.^35^ Briefly, ammonia (4 mL) and ethanol (50 mL) were mixed to form a homogenous solution, and TEOS (4.5 mL) was then added, after 1.5 h magnetic stirring at room temperature, a silica particulate sol was formed. The average diameter of silica nanoparticles (NPs) was measured by a particle sizer, which is around 200 nm (Fig. S1, ESI†).

### Acid–base stability tests

The coated fabric was immersed in strong acid (H_2_SO_4_, pH = 1) or base (NaOH, pH = 14) solution at room temperature for 5 hours. The immersed fabric was then rinsed with water and dried at 130 °C for 30 min.

### Preparation of FAS/FA-PEG-phosphate solution

0.15 g FAS was added to 30 mL FA-PEG-phosphate aqueous solution (3%, wt/v). After magnetic stirring for 30 minutes, a homogeneous coating solution was obtained.

### Coating treatment

Cotton fabric was immersed in the silica particulate solution for 1 minute to apply silica nanoparticles onto the fabric. The treated fabric was then dried at room temperature, followed by immersion in the FAS/FA-PEG-phosphate solution for 1 minute, and finally dried at 130 °C for 30 minutes.

### Abrasion resistance test

The abrasion resistance was tested using the Martindale method according to ASTM D4966. The test was performed with a commercial Martindale abrasion tester (I.D.M Instrument Design & Maintenance). In our experiment, the untreated fabric was used as the abradant. 9 kPa of pressure was employed.

### Characterizations

The diameter and distribution of silica particles were measured by a particle sizer (Zeta Sizer, Nano series). ^1^H, ^19^F and ^31^P NMR spectra were taken on Bruker Advance 400 NMR spectrometer. SEM images were captured using a SEM Supra 55VP operated at an acceleration voltage of 5.0 kV. AFM (Asylum Research) was used to measure surface roughness. A transmission electron microscope (TEM, JEM-200 CX JEOL, Seike Instrument) was used to observe the coated fibers. CA measurements were carried out on a contact angle goniometer (KSV CAM 101) using liquid droplets of 5 μL in volume. All the CA values reported represent the mean of 5 measurements. FTIR spectra were recorded on a Bruker VERTEX 70 instrument in ATR mode at a resolution of 4 cm^−1^ accumulating 32 scans. XPS were collected on a VG ESCALAB 220-iXL XPS spectrometer with a monochromated Al Kα source (1486.6 eV) using samples of ∼3 mm^2^ in size. The X-ray beam incidence angle is 0° with respect to the surface normal, which corresponds to a sampling depth of ∼10 nm. The obtained XPS spectra were analyzed by the CasaXPS software.

## Conflicts of interest

There are no conflicts to declare.

## Supplementary Material

RA-008-C8RA04645J-s001

RA-008-C8RA04645J-s002

RA-008-C8RA04645J-s003

RA-008-C8RA04645J-s004

RA-008-C8RA04645J-s005

RA-008-C8RA04645J-s006

RA-008-C8RA04645J-s007
