# Supplementary material for: Durable superoleophobic–superhydrophilic fabrics with high anti-oil-fouling property
Source: RSC Adv. 2018 Jul 30;8(47):26939–47. doi: 10.1039/c8ra04645j (PMC9083304; doi:10.1039/c8ra04645j)
Supplement: RA-008-C8RA04645J-s001 [file RA-008-C8RA04645J-s001.pdf]

Electronic Supplementary Material (ESI) for RSC Advances.
This journal is © The Royal Society of Chemistry 2018

Durable Superoleophobic-superhydrophilic Fabrics with High Anti-oil-fouling Property

Hua Zhou,[a] Hongxia Wang,[a] Weidong Yang,[b] Haitao Niu,[a] Xin Wei,[a] Sida Fu,[a] Shuai Liu,[c] Hao Shao,[a] Tong Lin*[a]*

a. Institute for Frontier Materials, Deakin University, Geelong, VIC 3216, Australia

b. Future Manufacturing Flagship, CSIRO, Clayton South, VIC 3169, Australia

c. School of Mechanical and Electric Engineering, Soochow University, 215000, China

Corresponding Authors' E-mail: hong.wang@deakin.edu.au; tong.lin@deakin.edu.au.

Electronic Supplementary Information

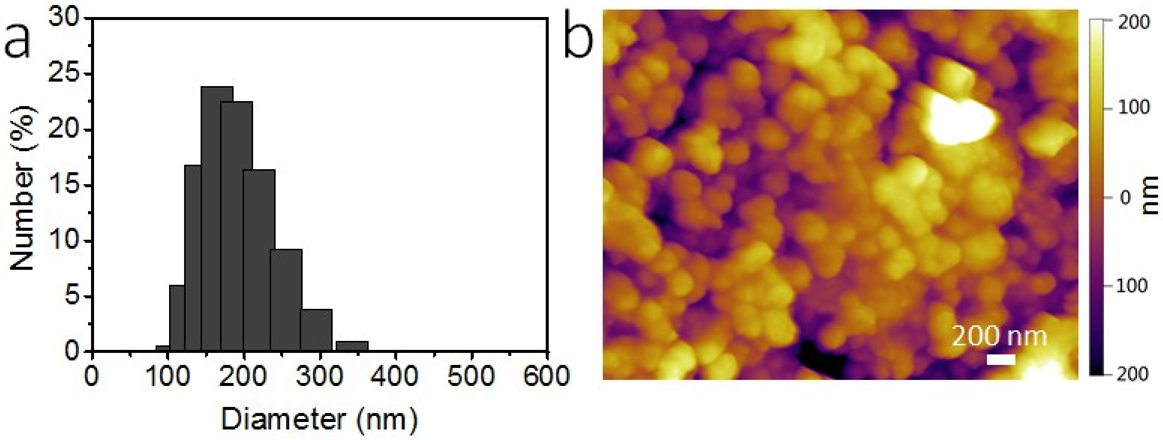

Fig. S1 (a) Histogram of size distribution of the silica nanoparticles (measured by particle sizer), (b) AMF image of the silica nanoparticles.

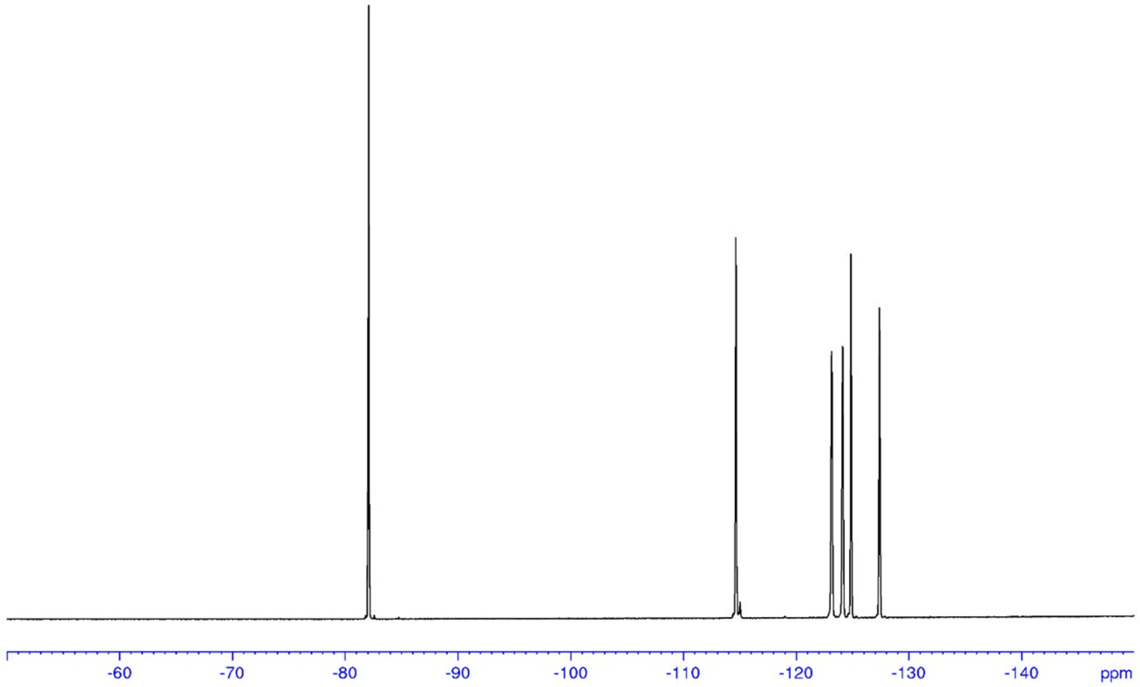

Fig. S2 19F-NMR spectrum of FA-PEG-phosphate.

1H NMR (400MHz, CDCl3): δ (ppm) = 2.2-2.5 (m, $-CF_2CH_2-$), 3.5-3.8 (m, $-OCH_2CH_2O-$), 4.1 (m, $-CH_2$-O-P-), 7.8(s, $O=P(OH)_2$). 19F NMR (376.5MHz, CDCl3): δ(ppm) = -81.8 (m, CF_3), -114.4 (m, $-CF_2CH_2-$), -123-125 (m, $-CF_2-$), -127.7(m, CF_3CF_2-). 31P NMR (162MHz, CDC_{l3}): δ (ppm) =1 ($-O=P(OH)_2$).

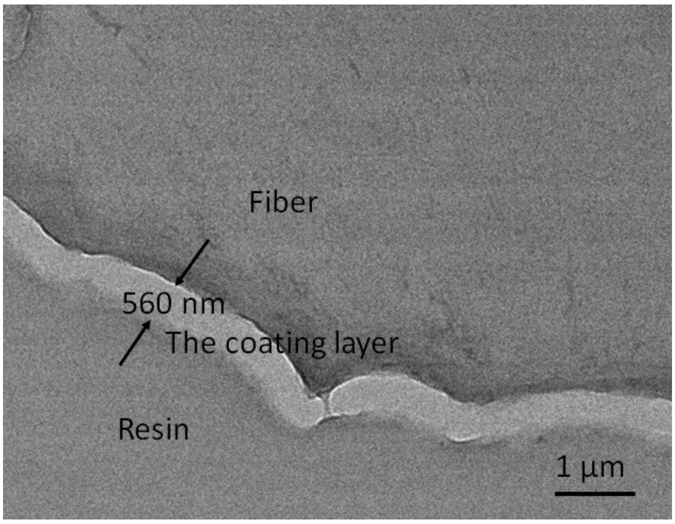

Fig. S3 TEM image of the coated cotton fiber.

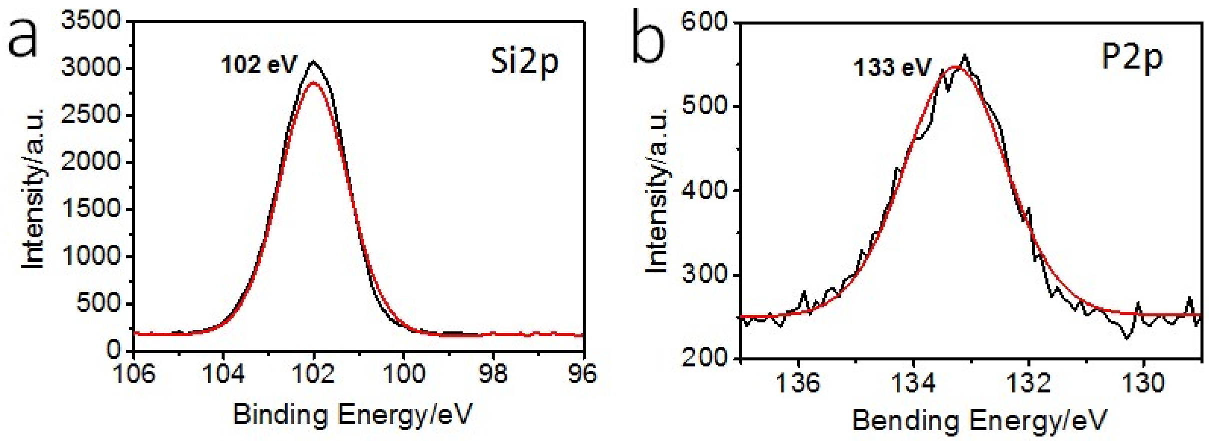

Fig. S4 XPS high resolution Si2p and P2p and curved fitted results of the coated cotton.

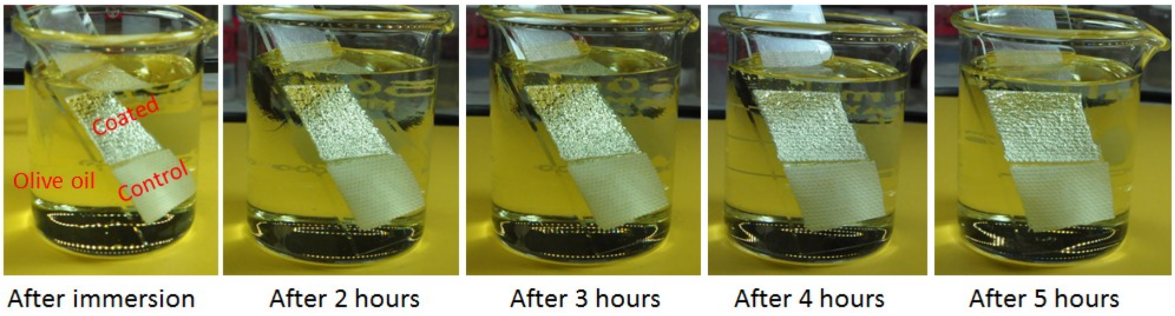

Fig. S5 Photo to show a piece of uncoated and a piece of coated cotton fabrics (as marked in the image) in olive oil for 5 hours.

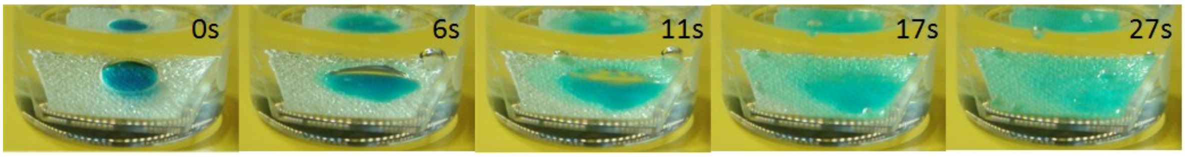

Fig. S6 Still frames taken from a video to show dropping blue dyed water (0.2 ml) on the coated fabric in olive oil.

When dropping 0.2 ml water on the fabric, which was fully immersed in olive oil (without the plastron layer), water spread into the fabric matrix in 27 seconds, indicating high affinity of the fabric to water.

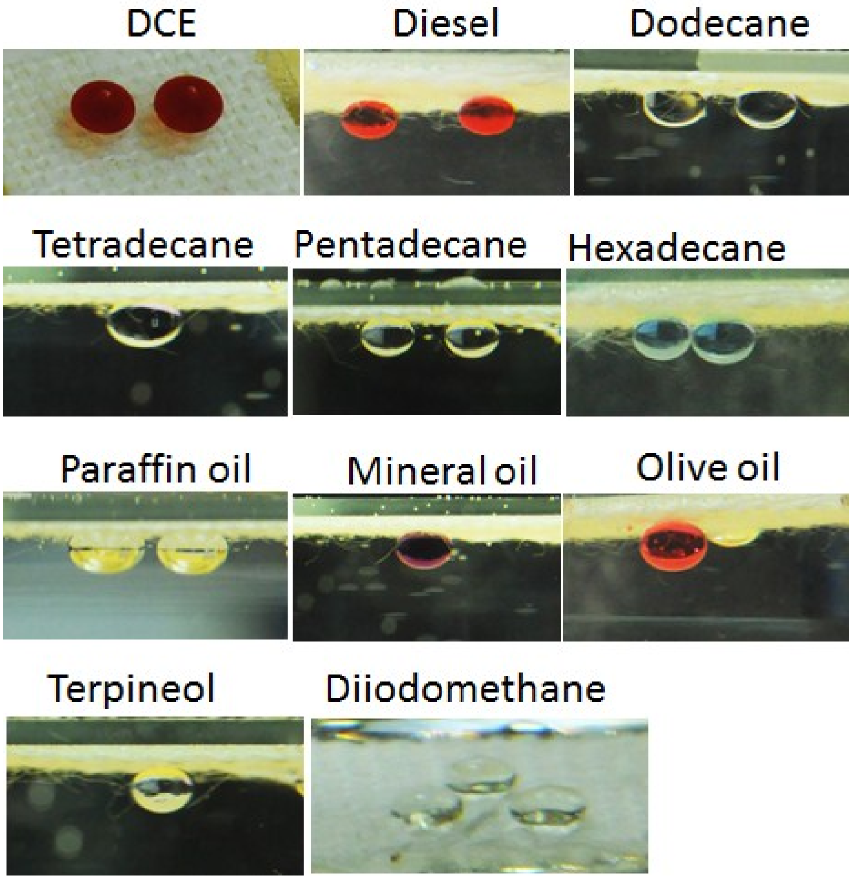

Fig. S7 Photos of different oil droplets on the coated cotton fabric in water state.

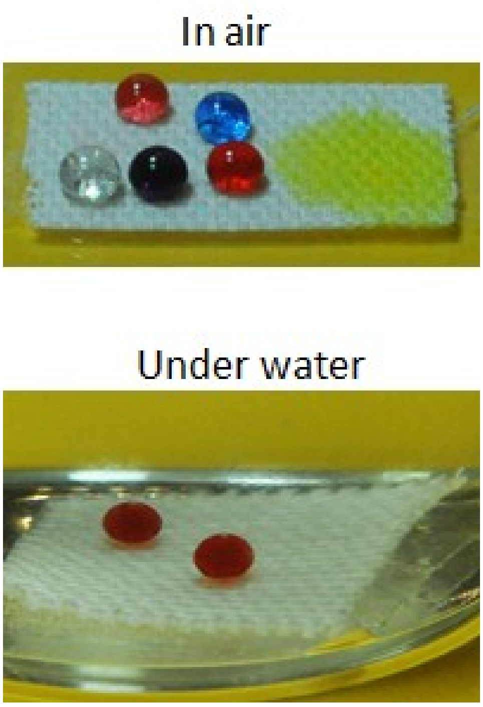

Fig. S8 (Top) Olive oil, mineral oil, paraffin oil, diesel, and hexadecane droplets stayed on the coated fabric. (Bottom) DCE droplets on the coated fabric immersed in water. The fabric was stored at ambient for 1 month.

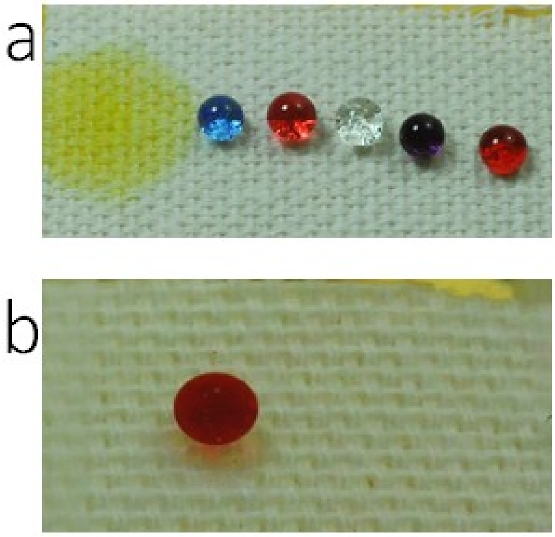

Fig. S9 Dropping different liquids on the coated cotton fabric: (a) in air (liquids from left to right are: water, hexadecane, diesel, paraffin oil, mineral oil, olive oil), (b) in water (liquid, DCE). The tests were conducted after immersing the fabric in water for 24 hours.

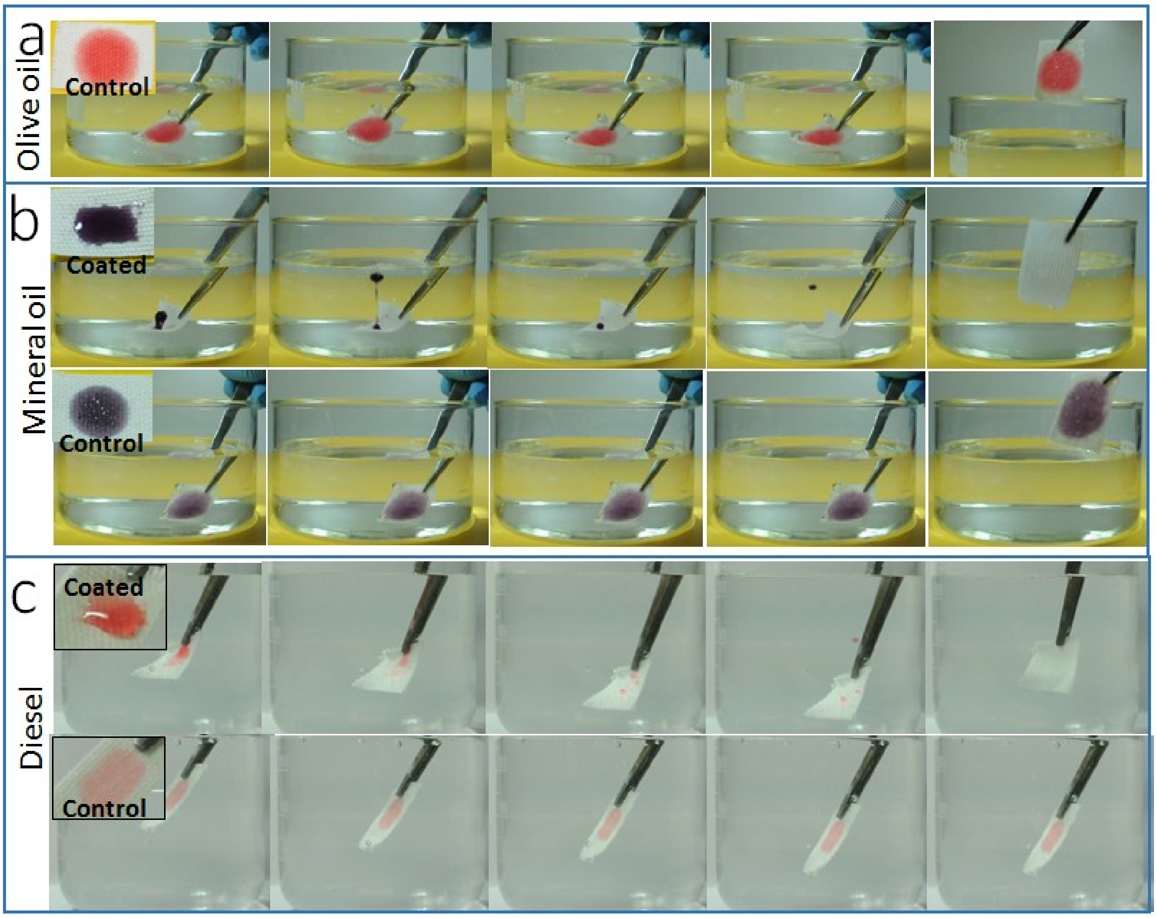

Fig. S10 (a-c) Still frames taken from videos showing contaminated cotton fabric (untreated) after immersing in water: (a) olive oil pre-contaminated, (b) mineral oil pre-contaminated, and (c) diesel pre-contaminated.

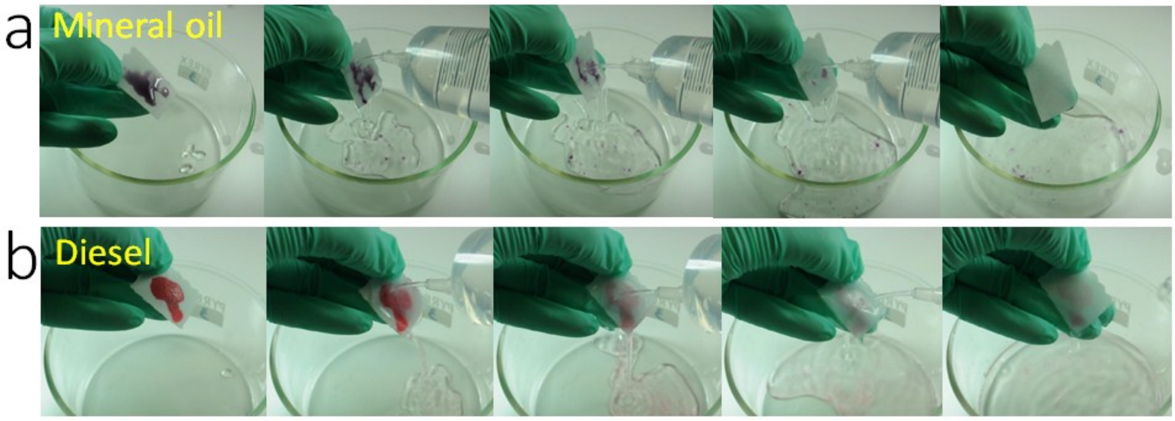

Fig. S11 Frames taken from videos to show: (a) rinsing the mineral oil fouled SHI-SOP fabric with water, (b) rinsing the diesel fouled SHI-SOP fabric with water.

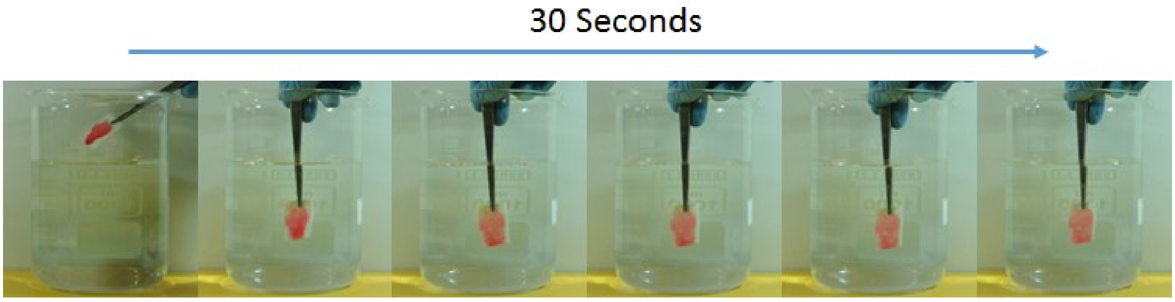

Fig. S12 Still frames taken from a video to show immersing an olive oil contaminated superamphiphobic cotton fabric in water.

Superamphiphobic cotton was prepared according to our previous paper (H Zhou, et al. *Adv. Mater. Interfaces*, 2015, 2, 1400559). Briefly, the cotton fabric was immersed in the PVDF-HFP/FAS solution for 1 min, after squeezing the redundant solution, the coated fabric was cured and dried at 130 °C for 30 min.

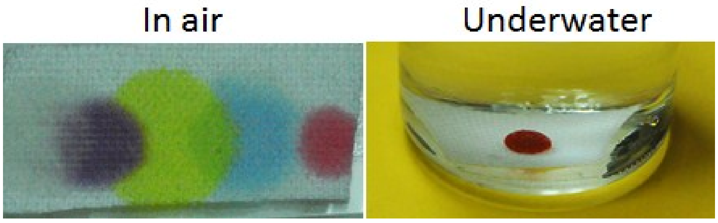

Fig. S13 Dropping liquids on uncoated cotton fabric in air and underwater. (In air: yellow water, purple mineral oil, red olive oil, blue hexadecane, and clear paraffin oil; In water: DCE)

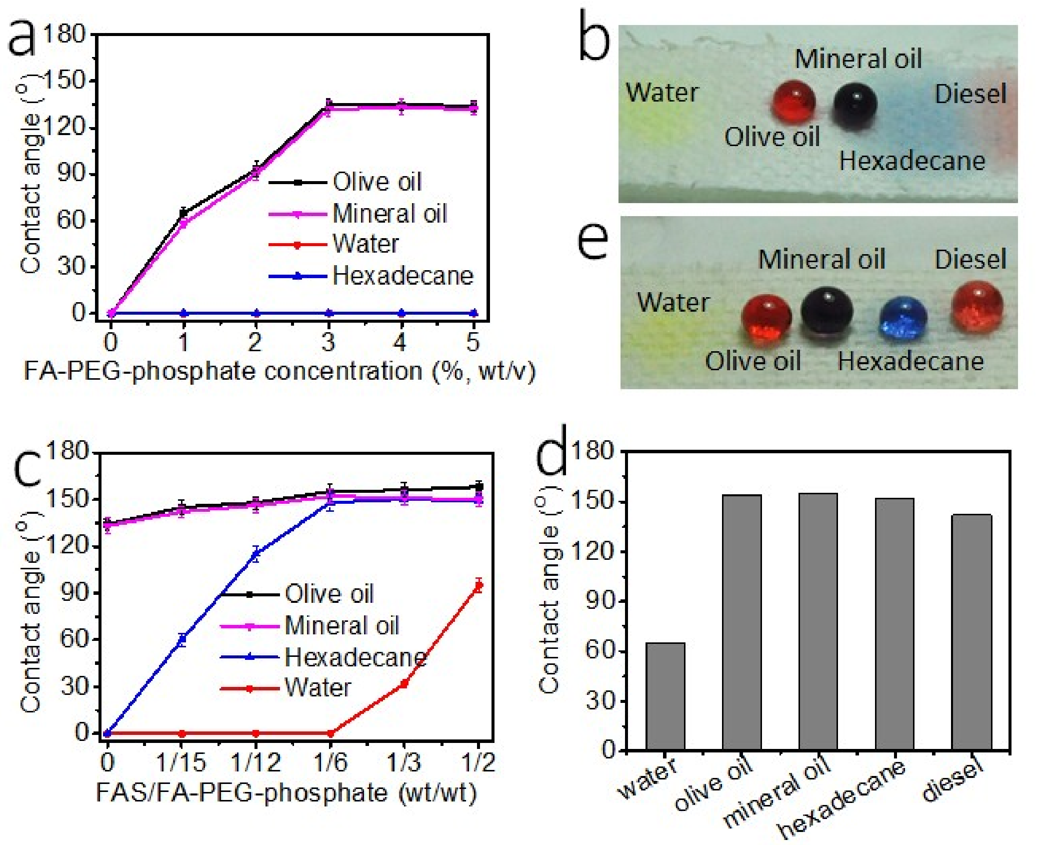

Fig. S14 a) Effect of FA-PEG-phosphate concentration on CAs for olive oil, mineral oil, hexadecane, and water of FA-PEG-phosphate coated cotton fabric, b) yellow water, red olive oil, purple mineral oil, blue hexadecane and light red diesel droplets on the 3% FA-PEG-phosphate coated cotton fabric, c) effect of the weight ratio of FAS and FA-PEG-phosphate on the CAs of the FAS/FA-PEG-phosphate coated cotton fabric (FA-PEG-phosphate concentration, 3.0% wt/v), d) water and oil droplets on the FAS/FA-PEG-phosphate (1/6, wt/wt) coated fabric surface, e) CAs of water, olive oil, mineral oil, hexadecane, and diesel of the hydrophobized silica NPs/FAS/FA-PEG-phosphate coated fabric.

When the concentration of FA-PEG-phosphate reached 3%, the FA-PEG-phosphate coated cotton showed the CA increased to 135° and 132° for olive oil and mineral oil, while the CA for water, diesel and hexadecane was 0° (see photo in Fig. S14a, b). Further increasing the FA-PEG-phosphate concentration did not improve the oil repellency.

Fig. S14c shows the effect of the weight ratio of FAS and FA-PEG-phosphate on the wetting property of the coated cotton fabric. When the FAS content increased from 0/1 to 1/6, the oil CA increased while the CA to water kept 0°. Further increasing the FAS portion in the coating solution, e.g. FAS/FA-PEG-phosphate from 1/6 to 1/2, the oleophobicity had slight increase, but the coated fabric showed an increased water repellency. The CA for water was 95° when the weight ratio was 1/2. To achieve a high level of surface oleophobicity as well as superhydrophilicity, a weight ratio of FAS/FA-PEG-phosphate=1/6 was used throughout this work.

Fig. S14d shows CA for water and oils when FAS modified hydrophobic silica nanoparticles were applied onto the fabric surface before the FAS/FA-PEG-phosphate coating treatment. However, when hydrophilic silica NPs were applied, the silica NPs/FAS/FA-PEG-phosphate coated fabric showed superhydrophilic-superoleophobic property (Fig. S14e).

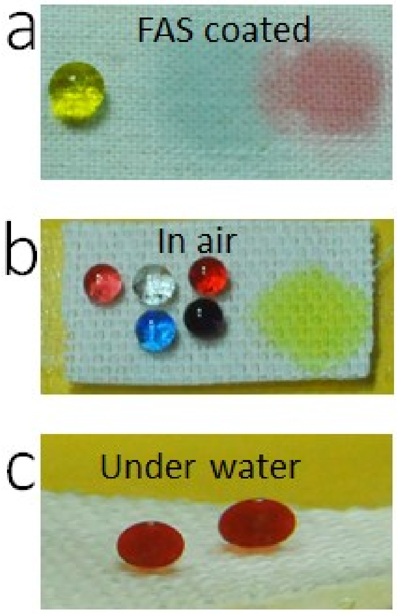

Fig. S15 a) Cotton fabric after coating treatment with 1% FAS (wt/v) in ethanol, b) light red diesel, red olive oil, clear paraffin oil, blue hexadecane and purple mineral oil on the coated cotton fabric after 1% FAS –pre-treatment, followed by silica NPs/FAS/FA-PEG-phosphate coating treatment, c) red DCE droplets on the FAS –pre-treatment and silica NPs/FAS/FA-PEG-phosphate treated cotton fabric in water.

To examine the effect of fabric wettability on coating treatment, we pre-treated cotton fabric with 1% (wt/v) FAS. The cotton fabric after FAS treatment showed superhydrophobic-superoleophilic property (Fig. S15a). When the hydrophobic cotton was further coated with hydrophilic silica NPs and then FAS/FA-PEG-phosphate, the coated cotton showed SHI-SOP in both air and underwater states (Fig. S15b, c). This indicates that the surface property of the fabric substrate has little effect on the final coating property.

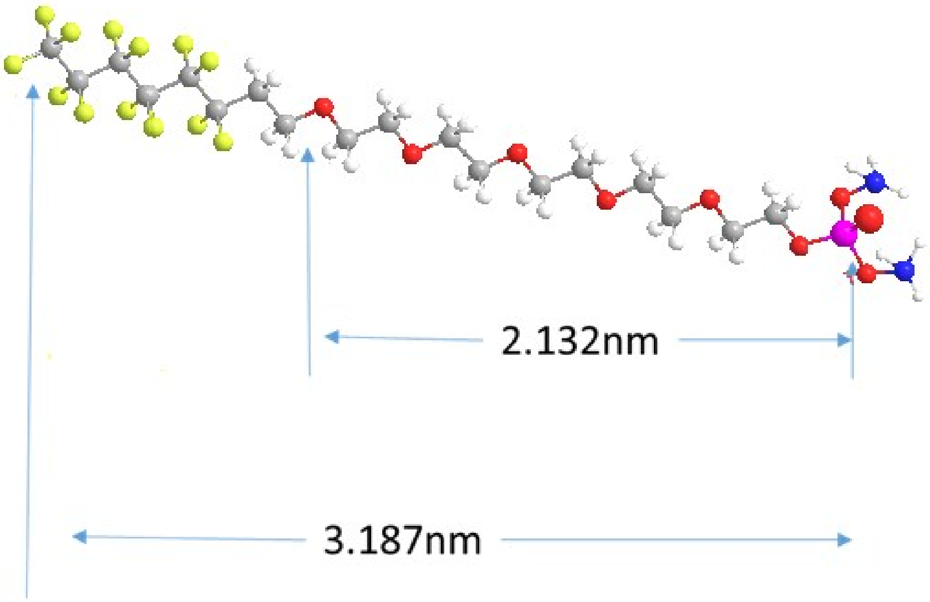

Fig. S16 Molecular size of FA-PEG-phosphate.

Table S1 Contact angle and water spreading time for the reported superhydrophilic fabrics

Superhydrophilic fabric	Water contact angle (°)	Spreading time (second)	References
Our work	0	1.58	
Wool fabric	0	1	Chen et al, *Langmuir*, 2009, DOI: 10.1021/1a903562h
Cotton	0	60	Jiang et al, *Applied Surface Science*, 258, 2012, 4888-4892
Polyester fabric	0	3	Xu et al, *Angew*, 2014, DOI: 10.1002/anie.201411283
Polyester fabric	0	—	Ashraf et al, *Journal of Colloid and Interface Science*, 394, 2013, 545-553
Cotton	0	—	Yang et al, *Advanced Materials*, 2013, 25, 1150-1154
Wool fabric	0	—	Pakdel et al, Applied Surface Science, 275, 2013, 397-402
Silk	0	14	Oh et al, RSC Advances, 2014, 4, 38966
PET fabric	0	3	Li et al, *Applied Surface Science*, 297, 2014, 147-152
Wool fabric	0	2	Mura et al, *Journal of colloid and Interface Science*, 456, 2015, 85-92
Cotton fabric	0	Several	Tang et al, *RSC Advances*, 2016, 6, 91301
Polyester fabric	0	—	Wang et al, RSC Advances, 2017, 7, 24374
Polyester	0	0.36	Li et al, *Applied Surface Science*, 427, 2018, 92-101
Cotton	0	—	Wang et al, *Separation and Purification Technology*, 195, 2018, 358-366

Table S1 summarizes the wetting results of the reported superhydrophilic fabrics. All the superhydrophilic fabrics show water contact angle of 0°. However, the time for water droplet to spread completely into the fabric varies. Some papers did not report the water spreading time. According to standard AATCC Test Method 79 “Absorbency of Textiles”, fabrics show superhydrophilicity if water droplet can spread into the fabric within 5 seconds. Combined with the above result, superhydrophilic fabrics can be defined as “water contact angle of 0° and if water droplet can spread into the fabric within 5 seconds”.

Table S2 CA of the coated fabric for the liquids with different surface tensions

Liquids	Surface tension (mN/m, 20 °C)	Viscosity (mPas, 20°C)	Contact angle (°)
Pentane	15.5	0.24	0
Silicon oil	21.5	9.30	0
Isopropanol	23.0	2.04	0
Diesel	25	3.03	145
Dodecane	25.35	1.34	147
Tetradecane	26.56	2.81	148
Pentadecane	26.9	3.73	150
Hexadecane	27.5	3.04	153
Paraffin oil	28	40	157
Mineral oil	30.8	20	159
Soybean oil	31.5	80.00	160
Olive oil	32.0	81.00	160
Terpineol	33.2	40	159
Diethylene glycol	44.8	35.7	162
Ethylene glycol	47.3	16.1	160
Tetraethylene glycol	48.0	58.3	161
Diiodomethane (DI)	50.8	2.76	158
Formamide (FA)	58.2	3.76	160
Glycerol	63.4	1412.00	163
Water	72.8	1.00	0

Table S3 CA_{uw} of the coated fabric for liquids with different surface tensions

Liquids	Surface tension (mN/m, 20 °C)	Contact angle (°)
DCE	28.2	170
Diesel	25	159
Dodecane	25.35	157
Tetradecane	26.56	154
Pentadecane	26.9	156
Hexadecane	27.5	155
Paraffin oil	28	157
Mineral oil	30.8	158
Olive oil	32.0	154
Terpineol	33.2	162
Diiodomethane (DI)	50.8	162
Water	72.8	0

Video S1 Coated fabric_dropping water on the fabric.

Video S2 Coated fabric_dropping water on the fabric which is immersed in olive oil.

Video S3 Coated fabric_immersing the olive oil-fouled fabric in water.

Video S4 Coated fabric_rinsing the olive oil-fouled fabric with tap water.

Video S5 Coated fabric_immersing the fully olive oil-fouled fabric in water.

Video S6 Control fabric_immersing the olive oil-fouled fabric in water.
